# Identification of Quantitative Trait Loci Relating to Flowering Time, Flag Leaf and Awn Characteristics in a Novel *Triticum dicoccum* Mapping Population

**DOI:** 10.3390/plants9070829

**Published:** 2020-07-02

**Authors:** Tally I.C. Wright, Angela C. Burnett, Howard Griffiths, Maxime Kadner, James S. Powell, Hugo R. Oliveira, Fiona J. Leigh

**Affiliations:** 1 The John Bingham Laboratory, NIAB, 93 Lawrence Weaver Road, Cambridge CB3 0LE, UK; aburnett@bnl.gov (A.C.B.); maxime.kadner@outlook.fr (M.K.); jsp532@york.ac.uk (J.S.P.); fiona.leigh@niab.com (F.J.L.); 2 Brookhaven National Laboratory, Upton, NY 11973, USA; 3 Department of Plant Sciences, University of Cambridge, Downing Street, Cambridge CB2 3EA, UK; hg230@cam.ac.uk; 4 Interdisciplinary Centre for Archaeology and Evolution of Human Behaviour, University of Algarve, 8005-139 Faro, Portugal; hroliveira@ualg.pt

**Keywords:** wheat, genetic diversity, tetraploid landraces, *Tritcum dicoccum*, QTL mapping, SNP genotyping, physiology

## Abstract

Tetraploid landraces of wheat harbour genetic diversity that could be introgressed into modern bread wheat with the aid of marker-assisted selection to address the genetic diversity bottleneck in the breeding genepool. A novel bi-parental *Triticum turgidum* ssp. *dicoccum* Schrank mapping population was created from a cross between two landrace accessions differing for multiple physiological traits. The population was phenotyped for traits hypothesised to be proxies for characteristics associated with improved photosynthesis or drought tolerance, including flowering time, awn length, flag leaf length and width, and stomatal and trichome density. The mapping individuals and parents were genotyped with the 35K Wheat Breeders’ single nucleotide polymorphism (SNP) array. A genetic linkage map was constructed from 104 F4 individuals, consisting of 2066 SNPs with a total length of 3295 cM and an average spacing of 1.6 cM. Using the population, 10 quantitative trait loci (QTLs) for five traits were identified in two years of trials. Three consistent QTLs were identified over both trials for awn length, flowering time and flag leaf width, on chromosomes 4A, 7B and 5B, respectively. The awn length and flowering time QTLs correspond with the major loci *Hd* and *Vrn-B3*, respectively. The identified marker-trait associations could be developed for marker-assisted selection, to aid the introgression of diversity from a tetraploid source into modern wheat for potential physiological trait improvement.

## 1. Introduction

Wild relatives and ancestral landraces of wheat offer a plethora of genetic diversity that could be utilised in breeding for improved yield and environmental stability in modern wheat. Domestication from progenitor species, coupled with modern breeding techniques, has narrowed the genepool of recent varieties [1]. Domestication of wheat took place around 12,000 BP and included diploid wheat as well as domesticated forms of the tetraploid *T. dicoccoides*, predominately *Triticum turgidum* subsp. *dicoccum* (*T. dicoccum*), commonly known as emmer wheat [2]. Modern wheat grown today mainly belongs to two species: the hexaploid *Triticum aestivum* (2n, 6x, 42 chromosomes; AABBDD) and the tetraploid *Triticum turgidum* subspecies *durum* (2n, 4x, 28 chromosomes; AABB), commonly known as bread and pasta wheats, respectively [3]. Bread wheat (*T. aestivum*) was formed through a chance hybridisation between *T. dicoccum* (AABB) and *Aegilops tauschii* (DD) [4,5,6].

Wild relatives and landraces of wheat are generally recognised to be an untapped genetic reserve for crop improvement [7]. Diversity from a tetraploid wheat background has already been used as a genetic resource for modern wheat, in particular for disease resistance [8,9] and abiotic stress tolerance [10,11]. However, introgression of beneficial traits from distant backgrounds can also include the unintentional ‘linkage drag’ of co-located genes linked to undesirable characteristics [12,13]. These issues can be reduced by the selection of a phenotype based on an associated DNA marker, so called ‘marker-assisted selection’ (MAS) [14], which can facilitate the introgression of quantitative trait loci (QTLs) from a donor background [15] and introgressions from an exotic background into modern wheat [16,17].

Selection solely for grain yield increases the risk of missing untapped genetic diversity from unimproved collections, which may be essential for future progress in breeding [18]. Trait-based selection overcomes this risk and may be particularly advantageous in capturing diversity from progenitor wheat linages. This approach suggests selection towards a crop ideotype [19], rather than traditional selection focused only on grain yield. When traits are screened for marker–trait associations, large populations are required for increased accuracy in estimating marker effects and improving the power of detection [14,20]. This means that (a) high-throughput techniques are required for rapid phenotyping of large mapping populations and (b) proxies are typically used for screening more complex physiological traits. Tetraploid wheat harbours diversity in photosynthetic [21] and drought tolerance characteristics [22]. However, these are time-consuming traits to screen and their study can be unfeasible in large populations.

Photosynthesis in the leaf and ear is a complex process that provides an important assimilate source for the developing grain [23,24]. The source determinant is controlled by the rate of photosynthesis and the total area of photosynthetic tissue [23], implying that selection for increased leaf or ear size may increase assimilate availability. There has been limited progress for breeding drought-tolerant varieties in a water-limited environment due to the complexity of the trait [25]. Reducing stomatal density has been shown to improve water use efficiency [26]. Alternatively, breeding for increased stomatal density may be more advantageous in water-rich environments, where flag leaf stomatal conductance has been linked to increased grain yield [27]. Trichome density has also been shown to be advantageous for drought tolerance in wheat [28] and could contribute to maintaining grain yield under drought conditions. Flowering time is a crucial determinant of drought avoidance [29] and is important for optimising yield potential within target environments [30]. Identification of candidate QTLs linked to such potential proxies for complex physiological processes in tetraploid landraces could aid the introgression of improved characteristics into modern wheat varieties.

The aim of this study was to create a novel tetraploid mapping population (*PS1*) through crossing two *T. dicoccum* landraces (Tios and dic12b). The *PS1* population was phenotyped in two trials and QTL mapping was completed for six traits which were potential proxies for photosynthesis and drought tolerance—flowering time, flag leaf width and length, awn length, flag leaf stomatal and trichome density. The results of the study should contribute to identifying candidate marker–trait associations for potential use in MAS to capture tetraploid landrace diversity.

## 2. Results

### 2.1. Phenotype Analysis

Trait heritability and mean values for individuals and parents for the traits measured in both trials are shown in Table 1. Significance testing between the parent samples is shown in Supplementary Tables S1 and S2. Across both trials, the parent Tios had significantly higher median values for the traits’ flowering time (F_T_) and flag leaf width (FL_W_), while dic12b had a significantly higher mean for awn length (A_L_) and median for trichome density (T_D_). Only for stomatal density (S_D_) did the variation between parents differ significantly across years. In 2017, dic12b had the significantly higher mean of 74.2 mm^−2^. However, Tios had the higher mean of 72.4 mm^−2^ in 2019, although the difference was not significant (*p* = 0.09). Across both trials, dic12b had a higher mean FL_L_ than Tios, although the difference was only significant in 2019. For A_L_, S_D_ and T_D_, generalised heritability (*H^2^*) was higher in the 2017 trial compared to 2019 (Table 1). In 2017, *H^2^* could not be calculated for FL_L_ due to the negative variance component associated with genotype in the model. Across both years, A_L_ was the most heritable trait. An example of A_L_ diversity within the population and between parents is shown in Supplementary Figure S1. In 2017, FL_W_ had the lowest calculated *H^2^* (0.36), whilst in 2019, the lowest heritability was observed for S_D_ (0.36).

**Figure 1:**
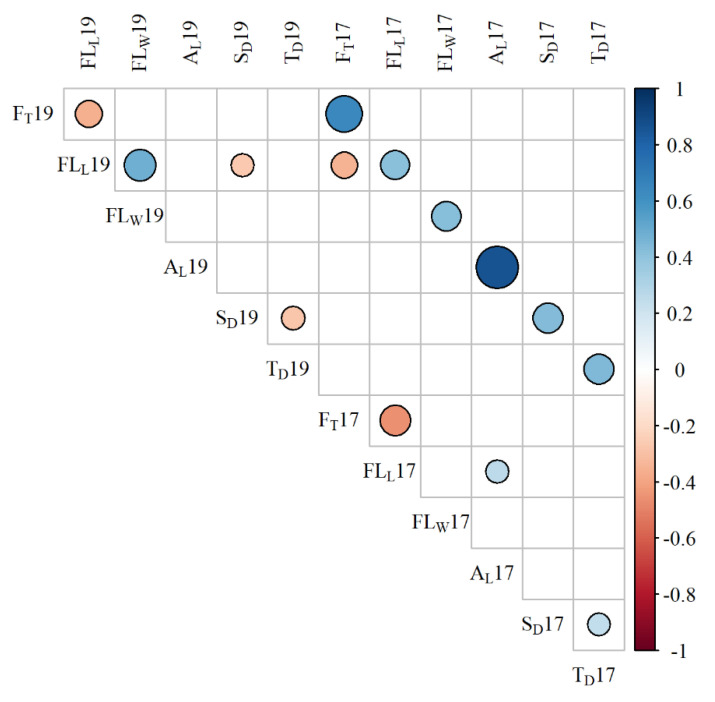
Pearson’s correlation matrix for the 6 traits measured across both trials. Where a square is blank, the Pearson’s product moment correlation test yielded a non-significant *p*-value (*p* > 0.01). A_L_ = awn length; F_T_ = flowering time; FL_L_ = flag leaf length; FL_W_ = flag leaf width; S_D_ = flag leaf stomatal density; T_D_ = flag leaf trichome density. The correlation matrix was formed in R using the package corrplot [31]. Plot font was edited using the R package extrafont [32].

Pearson’s correlation coefficient values between traits across both trials are shown in Figure 1. A significant positive correlation (*p* < 0.01) was observed for every trait when measured across the different years. A_L_ and F_T_ showed the strongest correlations between years (r = 0.86 and 0.64, respectively), while FL_L_ and FL_W_ showed the weakest correlations between years (r = 0.41 and 0.43, respectively). FL_L_ from 2019 was significantly negatively correlated with F_T_ from the 2017 and 2019 trials, while FL_L_ from 2017 was only negatively correlated with F_T_ in 2017. In 2019, FL_L_ also showed a significant negative correlation with S_D_, while a positive correlation was observed with FL_W_. Additionally, a negative correlation was found in 2019 between S_D_ and T_D_. This relationship was not observed in 2017, where S_D_ and T_D_ were significantly positively correlated. Finally, a positive relationship was also observed between FL_L_ and A_L_ in the 2017 trial.

### 2.2. Linkage Map

**Figure 2:**
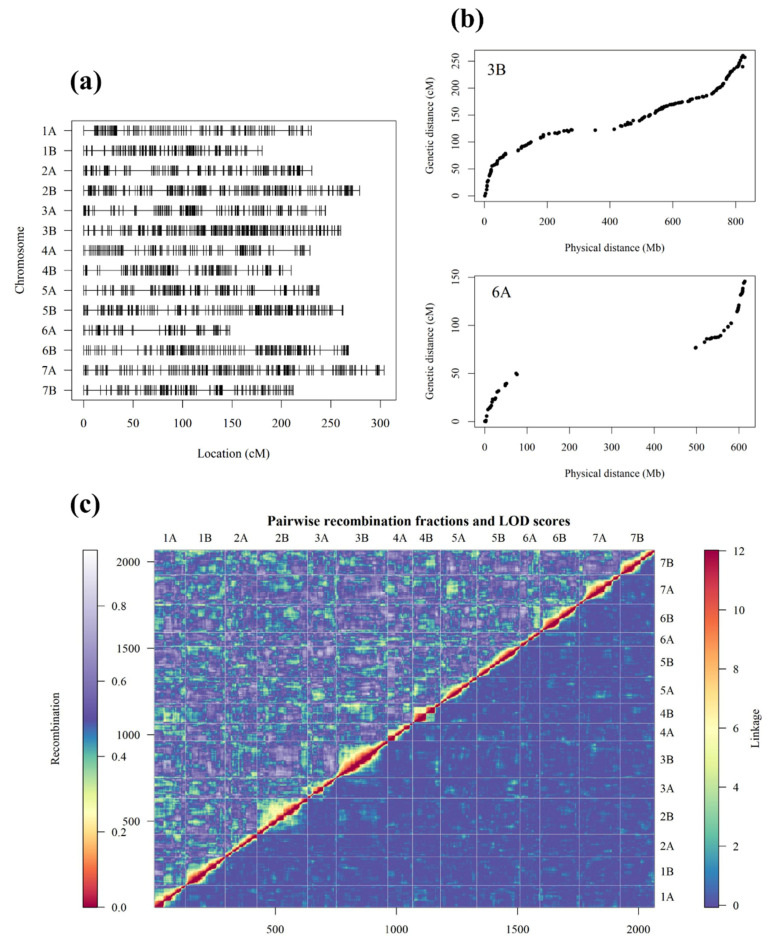
(**a**) The genetic map created for the tetraploid mapping population (*PS1*) showing marker spacing across 14 chromosomes, formed using the packages ASMap [33] and R/QTL [34] in R. The map was created using 2066 single nucleotide polymorphism (SNP) markers and 104 individuals. The vertical line on each chromosome represents the mapped position for each SNP. (**b**) The genetic mapped position of each SNP in the new map is shown relative to the physical position obtained through Basic Local Alignment Search Tool (BLAST+) searches of the RefSeq v1.0 wheat genome assembly [35] for two chromosomes: 3B and 6A, which showed the highest and lowest marker frequency, respectively. (**c**) A heat map showing the assembled *PS1* linkage map. The logarithm-of-odds (LOD) linkage between ordered markers is plotted in the bottom corner and pairwise recombination fractions between ordered markers is shown in the upper triangle, the heat map was created using the ‘heatMap’ function in ASMap [33]. Plot fonts were edited using the R package extrafont [32].

Using 104 genotyped *PS1* individuals, a new genetic linkage map was formed consisting of 2066 markers along 14 chromosomes (Figure 2a). Within each chromosome, the new genetic positions (cM) of the markers were correlated against the physical positions (Mb) for the same SNPs. Visual inspection of these plots indicated both appropriate ordering and orientation of each chromosome within the new genetic map. Examples of these plots are shown in Figure 2b for chromosomes 6A and 3B, which had the lowest and highest frequency of markers per chromosome in the linkage map (82 and 214 markers, respectively). Two heat maps were plotted as a further diagnostic of the new map: the pairwise recombination fraction and the logarithm-of-odds (LOD) linkage between ordered markers, shown in the top and bottom triangles of Figure 2c. Low recombination fractions and high LOD scores of closely neighbouring ordered SNPs indicated that the map was formed appropriately, this is shown through the high heat observed along the central diagonal of both heat maps in Figure 2c.

The total length of the new genetic map spanned 3295 cM, with an average spacing (between markers) of 1.6 cM. The largest spacing of 26.6 cM was found on chromosome 6A (Figure 2a). Chromosome length varied from 147.8 cM (6A) to 303.8 cM (7A). The lowest density of markers was found on chromosome 4A, with an average spacing (between markers) of 2.3 cM, and the highest density on chromosome 1B, with an average spacing of 1.1 cM. Across the new map, the B genome had a higher coverage of markers than the A genome, with 1185 associated markers across an average spacing of 1.4 cM compared to 881 markers with an average spacing of 1.9 cM. The complete genetic map formed using the *PS1* population is shown in Supplementary Table S3.

### 2.3. QTL Mapping

Results from the QTL mapping for both trials are shown in Table 2. In the 2017 trial, six candidate QTLs were identified for four traits across five chromosomes (2B, 4A, 5B, 6A and 7B). The percentage phenotypic variation explained by each QTL ranged from 12.3% (Q3_A_L__17) to 35.1% (Q5_A_L__17), and LOD scores ranged from 4.4 (Q8_FL_L__17) to 12.2 (Q5_A_L__17). The second highest LOD score was observed for Q9_F_T__17 (LOD = 7.1). The size of the 1-LOD support intervals, expanded to the nearest flanking marker, ranged from 7.2 cM for Q7_FL_W__17 to 124.5 cM for Q3_A_L__17. Of the QTLs found in the initial single genome scan for each trait, only Q7_FL_W__17 fell below the 0.05 alpha LOD threshold, but still passed the 0.1 threshold with an initial LOD score of 4.2. The additive effect for each QTL, shown in Table 2, indicated the effect of one dic12b allele (0.5 of BB) to the trait data. There was a positive additive effect of a dic12b allele at the QTLs: Q3_A_L__17, Q5_A_L__17 and Q7_FL_W__17, whilst there was a negative additive effect of a dic12b allele at the QTLs: Q2_FL_W__17, Q8_FL_L__17 and Q9_F_T__17. Using the residuals of F_T_ regressed on the FL_L_ data from 2019 produced no significant QTLs and, using the same approach with the 2017 data, there was little difference from the existing model found for Q9_FT_17. Using the residuals of the FL_L_ data regressed on F_T_ from 2019 produced no QTLs. However, using the residuals of the FL_L_ data regressed on F_T_ from 2017 produced a QTL on 6A (Q8_FL_L__17) that was not found using the raw trait data. The QTL had a LOD score marginally above the 5% alpha of 4.34 (LOD = 4.4). The parent dic12b had greater FL_L_ across both years, although a dic12b allele at Q8_FL_L__17 caused a negative effect on the trait (Table 2). However, the variation in FL_L_ between parents was reduced in the 2017 trial compared to 2019.

**Figure 3:**
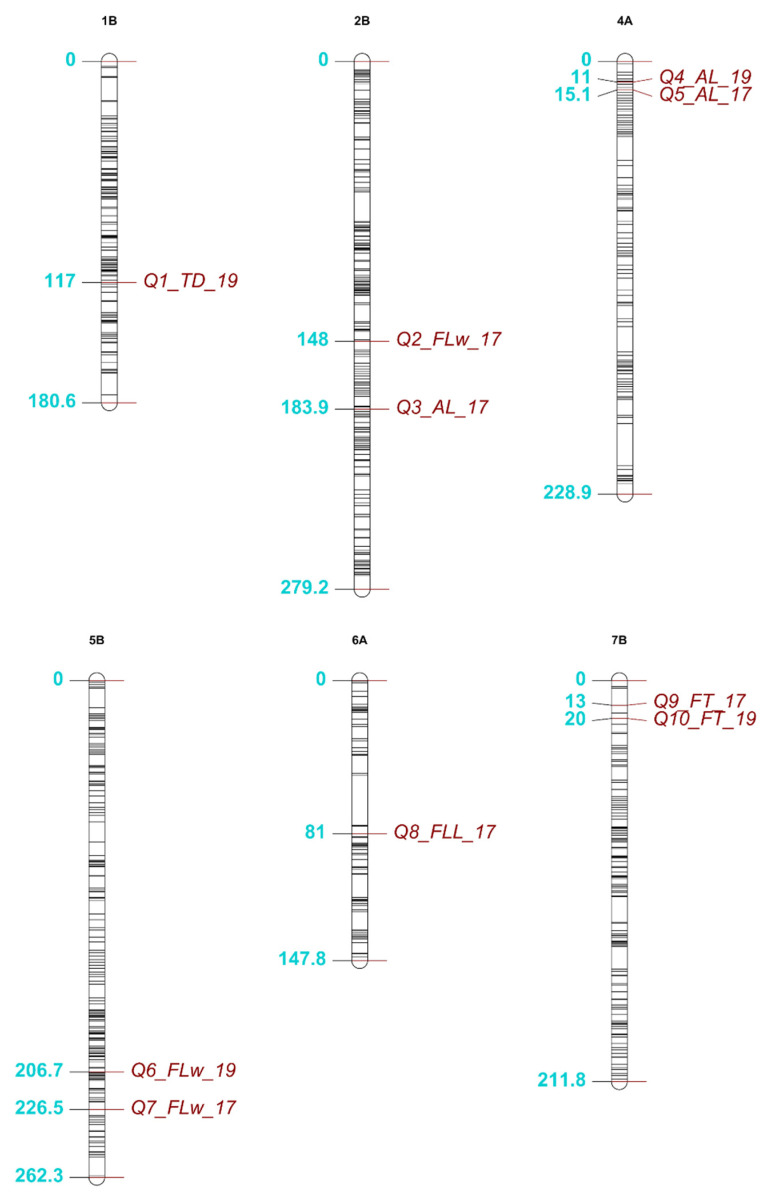
The distribution across the genome of the 10 quantitative trait loci (QTLs) identified across multiple years using the tetraploid mapping population (*PS1*). Six chromosomes are shown that carried QTLs for five of the six traits measured across both years, the black horizontal lines on each chromosome represent the mapped marker positions from the novel genetic linkage map. The location of each QTL is shown in blue alongside each label. The start and end of each chromosome is shown, and all positions are in cM. The figure was created using the R package LinkageMapView [37].

In the 2019 trial, four candidate QTLs were identified for four different traits, located on four chromosomes (Table 2). In 2019, Q4_A_L__19 yielded the largest LOD score and phenotypic variation explained by a QTL (11.6 and 39%, respectively). The distribution of the QTLs mapped across the genome in both years is shown in Figure 3. Several QTLs collocated: Q4_A_L__19 was mapped to 11 cM on 4A whilst Q5_A_L__17 mapped to 15.1 cM on 4A in the 2017 trial. Q10_F_T__19 was mapped to 20 cM on 7B, which co-locates with Q9_F_T__17 in the 2017 trial which was mapped to 13 cM on 7B. Further evidence that consistent QTLs were found across multiple years for A_L_ and F_T_ was shown through the dic12b allele contributing a positive additive effect at both Q4_A_L__19 and Q5_A_L__17, while the dic12b allele at both Q10_F_T__19 and Q9_F_T__17 contributed to a decrease in F_T_. Additionally, Q6_FL_W__19 mapped to 206.7 cM on 5B, was likely to be the same QTL as Q7_FL_W__17 mapped to 226.5 cM in 2017, where the dic12b allele contributed a positive effect on the trait for both QTLs. A single borderline QTL was found for T_D_ in 2019 (Q1_T_D__19) with a LOD score of 4.4, which was only slightly higher than the LOD 5% alpha threshold of 4.34.

The physical positions of the peak SNPs and 1-LOD support interval flanking markers for each QTL are listed in Table 3. As with genetic positions (Table 2), the largest physical 1-LOD support interval was found for Q3_A_L__17 (653 Mb), whereas the smallest physical interval (17.29 Mb) was found for Q9_F_T__17 (Table 3). The peak physical marker positions of the QTLs for A_L_ mapped to 4A across different years (Q4_A_L__19 and Q5_A_L__17) were relatively distant. However, the physical 1-LOD support intervals for each QTL were similar: 40 to 103 Mb for Q4_A_L__19 and 47 to 110 Mb for Q5_A_L__17. The peak marker was identical for Q9_F_T__17 and Q10_F_T__19. However, the peak markers for the QTLs mapped across different years for FL_W_ on 5B were 52 Mb apart.

## 3. Discussion

Typically, a polygenic trait will follow a relatively normal distribution, which was the case for all traits except trichome density (T_D_). In bread wheat, awn development is considered to be determined by a few major loci [38], while the flowering time pathway is considered to be more complex [39]. However, transgressive segregation appeared greater in awn length (A_L_) than flowering time (F_T_) across both trials (Supplementary Figure S2). Transgressive segregation was still observed for every trait, indicating the presence of complementary alleles inherited in the progeny from both parents [40]. This combination of complementary alleles, leading to progeny with extreme phenotypes, would be useful for trait-specific ideotype selection, and hence genetic improvement of these traits in breeding programmes. Furthermore, high heritability was found consistently for three of the six traits measured (A_L_, F_T_ and T_D_) and moderate heritability was observed for the remaining traits (stomatal density (S_D_), flag leaf length (FL_L_) and width (FL_W_)) in at least one trial. The high to moderate heritability observed for the traits indicated high genetic variability within the population and suggested that there were likely to be underlying QTLs linked to phenotypic variation (particularly for A_L_, F_T_ and T_D_).

For A_L_ and F_T_, there were major loci segregating in the population, which meant that these QTLs were easily detectable. For both S_D_ and FL_L,_ there was no consistent significant variation observed between the parent lines across trials. Furthermore, low *H^2^* was observed for S_D_ in 2019 and no *H^2^* estimate could be obtained in 2017 for FL_L_, due to a negative variance term associated with genotype. These observations signified that no major loci were segregating for these traits and that the variation observed may be a result of the environment. However, across the two trials, each of these traits were significantly correlated, suggesting the presence of some genotypic effects controlled by minor QTLs. This was supported by the 2017 trial, where there were moderate *H^2^* observed for S_D_ (Table 1) and a minor QTL found for FL_L_ (Table 3). It was probable that the relatively small population size used reduced the power of detection of further minor QTLs. The distribution of the T_D_ data before the log transformation suggested that there may have been a major loci controlling the trait, further supported by the consistent significant variation between parents across trials and the high *H^2^* observed. However, only a single minor QTL was found for T_D_ in 2019 that contributed a small effect (17%) to the observed phenotypic variation, potentially due to poor marker coverage surrounding the segregating loci. There have been limited investigations into QTLs controlling T_D_ in wheat. The negative correlation observed between T_D_ and S_D_ in 2019 indicated that the T_D_ variation may have been influenced by physiological trade-offs with other phenotypes.

One of the difficulties in ideotype design and trait-based selection are the potential trade-offs or compensations between characteristics [18,19]. This was apparent in this study from a number of negative correlations observed in the trials (Figure 1), such as between FL_L_ and F_T_, which was observed over multiple years. No QTLs were found for S_D_ in either trial. In 2019, the Tios parent had a noticeably shorter flag leaf than in 2017, and as FL_L_ negatively correlated with S_D_, this may have caused an increase in S_D_ on the flag leaves of the Tios line. T_D_ correlated negatively with S_D_ in 2019, which could suggest that the partitioning of more assimilates towards a higher stomatal density per unit leaf area may cause a reduction in trichome number. This negative relationship could be beneficial for breeding in a water-rich environment, where stomatal density would be favoured over trichome density for increased CO_2_ capture, regardless of water loss. However, a significant positive correlation was observed between the traits in 2017 (Figure 1), suggesting that environmental factors may have had the greatest influence on the relationship. There were trait relationships reflecting assimilate investment into both the flag leaf and the ear: FL_L_ and A_L_ were positively correlated in the 2017 trial. Investment into ear area has been previously linked to reductions in flag leaf area [41], so the combination of increased organ size found in the present study may be promising for breeding high yielding lines [42]. Marker–trait associations, such as the ones found in the present study, would be useful tools in the selection for maintaining favourable trait combinations.

Three consistent QTLs were found over multiple years linked to three traits (A_L_, F_T_ and FL_W_). The most significant of these was linked to A_L_ and mapped to 4A, where the longer awned parent (dic12b) allele increased A_L_ (Table 2). Although not yet cloned, there is a known gene on the short arm of 4A that causes awn shortening and bending in common wheat, called the *Hd* locus [43]. In a hexaploid wheat recombinant inbred line population, Yoshioka et al. [38] identified three major QTLs related to awn length which corresponded to the three known inhibitor genes: *Hd*, *B1* and *B2* (located on 4A, 5A and 6B, respectively). Yoshioka et al. [38] found that the QTL on the short arm of 4A corresponded to the *Hd* locus located at 22 cM and explained 23.1% of the phenotypic variation. In another hexaploid wheat population, Sourdille et al. [44] mapped two QTLs relating to awn length, one of which segregated with the *Hd* gene. The genetic control of awn length and presence is less studied in *T. dicoccum*. However, in a *T. dicoccum* and *T. durum* mapping population, Buerstmayr et al. [45] found a QTL on the short arm of chromosome 4A that was linked to A_L_ and they concluded that it may correspond to the *Hd* locus. Although the confidence interval identified in the present study was relatively large (Table 2 and Table 3), it is probable that the 4A QTL was segregating with the major *Hd* locus. The second QTL linked to A_L_ was found on chromosome 2B in the 2017 trial, the same QTL was also apparent in 2019 but fell just under the significance thresholds used and was thus not reported. Other studies have also found QTLs controlling A_L_ aside from the three known inhibitor gene loci [46,47]. However, there is no previous evidence in the literature supporting the 2B QTL found in the present study.

The F_T_ QTL mapped to 7B had the smallest physical 1-LOD support interval of the QTLs mapped across multiple years (Table 3). Flowering time control in tetraploid wheat is less studied than in hexaploid wheat [48]. In hexaploid wheat, the genetic control of F_T_ is complex and controlled by three signalling pathways: vernalisation (*Vrn* genes), photoperiod response (*Pdp*) and earliness per se (*Eps*) [39,49]. Würschum et al. [48] studied 328 European durum genotypes and found six QTLs linked to heading time, with their hits on 2B and 7B corresponding to the known genes *Ppd-B1* and *Vrn-B3*, respectively. The physical position of the 7B QTL in Würschum et al. [48] fell within the physical 1-LOD support interval of the QTL mapped on 7B in the present study. Variation in *Vrn-B3* has been found to be prolific in durum wheat [50] and in *T. dicoccum* [51]. *Vrn-B3* is considered to be linked exactly to the gene in Arabidopsis Flowering Locus T (*FT*) and is known to have some control on flowering time [52]. Therefore, although both the mapping parents (Tios and dic12b) had no vernalisation requirement, it is probable that this major locus was linked to flowering time in the *PS1* population.

A QTL was only identified for FL_L_ in 2017 once the effect of F_T_ had been removed. As no additional QTLs were identified for F_T_ once the FL_L_ effect had been removed, it was probable that an earlier flowering time influenced flag leaf growth. F_T_ is known to be an important factor in determining many physiological traits [53]. A stable QTL was found for FL_W_ towards the end of 5B (Table 2). Other studies have identified a considerable number of environmentally stable QTLs for FL_W_ and FL_L_ in hexaploid wheat [54,55]. However, there has been limited investigation into QTLs linked to flag leaf area in a tetraploid wheat background. Yang et al. [56] did identify a QTL linked to FL_W_ in a hexaploid population on the long arm of 5B which was stable under multiple environments, although they mapped 15 QTLs for FL_W_ in total. This evidence indicates that FL_W_ in wheat is probably controlled by many minor QTLs, including the 5B QTL identified in the present study.

The first step of MAS application in breeding programs is the identification of candidate QTLs for marker–trait associations, which has been achieved in the present study for five hypothesised proxies that relate to photoassimilate supply or drought tolerance. Compared to modern wheat, there has been relatively limited previous investigation into the genetic control of this combination of traits in tetraploid landraces. The genotyped *T. dicoccum* mapping population produced in this study is a useful tool for the research and breeding community and could be employed further for the identification of marker–trait associations in a diverse background. Improved resolution of QTL locations in this study would be needed before these markers could facilitate the introgression of diverse physiological characteristics into modern wheat from a tetraploid source. Building on the present study, this goal could be achieved by fine mapping approaches or by increasing the number of individuals in the mapping population.

## 4. Materials and Methods

### 4.1. Plant Material

A tetraploid mapping population (*PS1*) was created by crossing two *T. dicoccum* landraces, shown in Supplementary Figure S1. The landraces were taken from a collection described in Leigh et al. [57], and the maternal parent ‘Tios’ and the paternal parent ‘dic12b’ originated from Spain [58] and Iran, respectively. Parental samples were taken from single founder plants to reduce inherent genetic heterogeneity in these landraces. F2 seed of 200 individuals originating from six F1 plants was sown into 96-well trays and grown in glasshouses at Park Farm, Cambridge. Individuals were taken through a single seed decent program to the F5 generation from October 2014 to March 2017. At each generation, flowering ears were covered with a clear cellophane bag to prevent outcrossing and promote self-fertilisation. By the F5 generation, the population size had reduced to 101 due to germination failures and sterility, which may have been related to the poor adaption of the landraces to the glasshouse environment. As a result, F4 seed was included in a field trial with the F5 seed to increase the population size. Of the 120 individuals that reached maturity in the trial, 87 and 33 were from F5 and F4 seed, respectively. Seed was hand threshed from ears collected from this trial and 108 individuals were advanced to a further generation (F5 or F6) in an outdoor 5 L pot trial sown on 29 April 2019.

### 4.2. Trials and Phenotyping

The *PS1* population was phenotyped in two trials conducted in outside areas. Firstly, a mix of F4 and F5 plants were grown in a field trial at the New Ornamentals Field, Park Farm, NIAB, Cambridge. Seeds were hand-sown into two replicated 25.5 m^2^ plots (8.5 × 3 m) on 24 March 2017. Each plot had nine 8.5 m long rows spaced every 30 cm. The 1st and 9th row and the 25 cm at the start and end of each row was sown with seed of hexaploid bread wheat variety Paragon to create a buffer and to reduce edge effects. The 2nd, 5th and 8th rows were not sown with any seed, in order to aid accessibility for phenotyping. The 3rd, 4th, 6th and 7th rows were organised into a randomised incomplete block design with 5 blocks per plot. Within each block, there were 32 mini-plots (30 mapping individuals and the 2 parents). Each mini-plot was a 20 cm row segment, consisting of a label and 3 seeds sown 5 cm apart. The trial was fertilised with one application of ammonium nitrate (34.5% N) at a rate 434 kg ha^−1^, in order to achieve a universal N application of 150 kg/N, and treated with insecticide and fungicide in two applications. The trial was manually irrigated. Every plant in the trial was individually staked to avoid lodging. Non-invasive measurements were made on the flag leaves of the primary tiller from the first individual in each mini-plot to reach growth stage (GS) 61 [59]. Leaf impressions were taken from the second plant to reach GS61. If no second plant germinated across the trial for a particular line, the impression was taken on the flag leaf of a secondary main tiller of the first plant. Only one of the two replicated plots was used for the measurements; however, if an individual failed to grow in the 1st plot it was measured from the 2nd plot. All parent mini-plots were measured across the trial in order to test for potential differences between plots.

Seed was harvested from the 2017 field trial and sown in a subsequent 2019 trial. Six seeds of the same individual were sown in one 5 litre pot on 4 April 2019 in an unheated glasshouse and thinned on germination to leave three viable seedlings. Pots were transferred to an outside trial area at Park Farm, NIAB, Cambridge, on 24 April 2019, where they were laid out in an incomplete block design with two replicate pots per individual, 20 replicates of each parent and a block size of eight pots. The design consisted of 15 columns, with the outer columns containing pots filled with Paragon plants to act as a buffer. The 2nd, 5th, 8th, 11th and 14th columns were left as 0.5 m walkways to aid accessibly for phenotyping. All plants were grown in an 80% peat-reduced and 20% bark compost mix, with a slow release fertiliser incorporated into the mix. A single fungicide application was made. Within each pot, measurements were taken from primary tillers of the plant furthest from the walkway at GS61. All pots were connected to an automated drip irrigation system ensuring even water availability across the trial.

For both trials, measurements of flag leaf length (FL_L_), width (FL_W_), awn length (A_L_) and the number of days from sowing to flowering time (GS61, F_T_) were recorded. An abaxial leaf impression from halfway up the flag leaf was made onto a microscope slide using Loctite Power Flex Ehyl-2-Cyanocrylate (Loctite, Düsseldorf, Germany). A uniform mesophyll region from each slide was imaged using a Leica DM2500 Optical Microscope (Leica Biosystems, Nussloch, Germany) at 5x magnification. Stomata and trichomes were counted from the 5x images in three different 0.3 mm^2^ overlaid grid squares and the mean values were taken, enabling stomatal and trichome density to be estimated per mm^2^ (S_D_ and T_D_, respectively). Only fully developed trichomes were included in the counts, where the trichome appendage was clearly visible.

### 4.3. Trial Analysis

Many of the seeds across the mini-plots in the 2017 field trial failed to germinate. However, for all traits except F_T_, there was no evidence that the germination heterogeneity influenced the trait (data not shown). For F_T_, there was strong evidence that the germination heterogeneity influenced the trait. F_T_ trait data were therefore regressed on the germination scores and residuals were extracted from the model. The residuals were used as the adjusted trait data for QTL mapping. As the majority of the data in the 2017 field trial came from single replicates, no adjustment for spatial trends were made and raw data were used in the QTL mapping for the five remaining traits.

The increased use of replicates in 2019 enabled all traits to be adjusted for spatial effects, as implemented via the R package SpATS [60]. After adjusting spatial trends through two-dimensional P-spline modelling, best linear unbiased estimations (BLUE) per line were extracted and used as phenotypic data in the QTL mapping. Frequency histograms were plotted in R with the package ggplot2 [61] and showed that T_D_ data from both years were not normally distributed, as the most frequent observed T_D_ was close to 0. To avoid violating underlying assumptions made through statistical testing, T_D_ data from both years were expressed to the log10. A negative correlation was found between FL_L_ and F_T_ in both trials. To assess marker–trait associations independent of this relationship, each trait was regressed on the other within each year. The residuals were extracted from each model and tested as phenotypic data in the QTL mapping, and the results were compared using the uncorrected trait values.

For the 2019 trial, generalised heritability (*H^2^*) was calculated using the ‘getHeritability’ function within SpATS, which implements a calculation suggested by Oakey et al. [62]. For the 2017 trial, *H^2^* was also calculated on a line mean basis, implemented by the VHERITABILITY procedure in Genstat [63], which employs a calculation of *H^2^* from Cullis et al. [64].

### 4.4. Genotyping

DNA was extracted from seven-day-old seedlings of 115 F4 *PS1* individuals and parental lines following a modified Tanksley 96-well-microprep extraction method [65]. DNA samples were sent to Bristol University for genotyping using the Axiom 35k SNP Wheat Breeders’ Genotyping Array [66]. This array has previously been used for identifying QTLs and population structure in tetraploid wheat [67,68].

Marker allele calls were made using the Affymetrix Axiom Analysis Suite (www.thermofisher.com) with some alterations from the default parameters to account for the ploidy and diversity of the material: a dish quality control (DQC) threshold of 0.8, a genotype call rate threshold of 96% and a quality control (QC) call rate of 96% were used for the SNP calling. All markers with a call rate between 96% and 98% were manually inspected and recalled or disregarded appropriately. Markers named as ‘no minor homozygote’ were manually inspected and the missing homozygote classes were recalled where possible. Additionally, markers with a QC call rate between 95% and 96% were manually inspected and where clustering was still well defined, the markers were included in the analysis.

Further quality control and analysis was completed using R (version 3.5, [69]) in RStudio [70]. Markers that were monomorphic between the parents or heterozygous within a parental line were removed. Based on genotypes, Euclidean distances between individuals were calculated and plotted through principal coordinate decomposition, which was implemented by the R package ape [71]. Two pairs of individuals appeared to be more closely related than expected, suggesting possible out-crossing or mixed samples at some stage of the single seed decent program. One individual from each of these pairs was removed from the analysis. Heterozygosity frequency in markers and individuals were plotted and visually inspected, and a single outlier genotype and markers with over 25% heterozygote calls were removed. ChiSquare tests were used to remove markers that differed significantly from the expected allele frequency (1:1) typical of Mendelian inheritance. *p*-values (*p*) from these tests were adjusted for multiple tests using a Bonferroni correction. After the QC, 4274 markers and 112 individuals remained in the dataset.

### 4.5. Genetic Linkage Map Formation and QTL Mapping

The R package ASMap (V - 1.0-4, [33]) was used for the map construction and remaining quality control. The ‘pullCross’ function was used to remove 2144 co-located markers. Using the ‘mstmap’ function, a numerical threshold value of *p* = 1 × 10 ^−12^ was set for linkage group clustering, which separated the markers into an initial 25 different linkage groups. Using a one-tailed test of a Poisson mean and a Bonferroni adjustment for multiple testing, eight individuals were removed with a significantly different (*p* < 0.01) crossover number from the population median. Single markers that formed a unique linkage group or which had more than two double crossovers were removed from the map. Following Broman [72], genotype error rate was estimated through maximum likelihood as 0.0025 and genotypes with high error logarithm-of-odds (LOD) scores were identified and removed (LOD > 6). Markers from the SNP array were all run through the Basic Local Alignment Search Tool (BLAST+) command-line applications [73] against the RefSeq v1.0 wheat genome assembly [35] using default parameters. All markers with a single hit to the A genome were selected and retained for analysis of the A genome regardless of other genome hits; similarly all markers with a single hit to the B genome were selected for analysis of the B genome, regardless of other genome hits. The newly formed linkage groups were then compared to the physical positions of the extracted markers from the BLAST+ results and the breeders’ array consensus map [66]: 28 linkage groups were merged and renamed appropriately. Marker physical positions were plotted against the mapped genetic position for each linkage group and 21 markers were dropped that were mapped to different physical and genetic chromosomes. Finally, heat maps for each chromosome were generated to validate marker ordering and check for linkage outside of the central diagonal. No issues were found through the heat maps and no changes were made. The completed map was formed from 2066 markers and 104 individuals.

QTL mapping was carried out for every trait using R/QTL (V - 1.44-9, [34]) and the map consisting of 2066 markers. The genetic marker data used is shown in Supplementary Table S4. Phenotype data was used from 111 and 108 individuals from the 2017 field trial and the 2019 pot trial, respectively. To avoid violating the underlying assumptions of the model and creating false positives, a late flowering outlier was removed from the 2019 F_T_ data before the QTL analysis was completed, the data point was a BLUE estimated from only a single replicate as the other replicate failed to grow. Following Broman and Sen [36], QTL genotype probabilities were calculated using the ‘calc.genoprob’ function with a step size of 1 cM. A two-stage QTL analysis was used. Firstly, a single genome scan was completed using the ‘scanone’ function. The LOD thresholds at significance levels of 5% and 10% (0.05 and 0.1 Alpha) were determined through running 5000 permutations within the ‘scanone’ function. If a QTL passed the determined 10% threshold, it was advanced to the second stage of analysis, where the functions ‘makeqtl’ and ‘fitqtl’ were used to fit a multiple-QTL model. For each trait, the function ‘addqtl’ was used to scan for additional QTLs, which were added to the model if the resulting LOD score from the function was over the initial 5% threshold. Where multiple QTLs were found, interactions between all QTLs in each model were tested for using the ‘addint’ function. QTL positioning was refined using the function ‘refineQTL’ and 1-LOD support intervals were determined using the function ‘lodint’ and were expanded to the nearest marker. Haley–Knott regression was implemented for all mapping functions. The physical positions on IWGSC Refseq v1.0 for all peak and 1-LOD support interval flanking markers were obtained from the previous BLAST+ results.

## 5. Conclusions

A novel *T. dicoccum* mapping population was created and QTLs were successfully identified using a map that we created consisting of 2066 SNPs. Candidate QTLs for A_L_ and F_T_ were mapped consistently over different trials and were likely linked to the major locus *Hd* and *Vrn-B3*. Novel candidate QTLs were potentially identified for FL_W_, T_D_ and FL_L_ but further work is needed for validation. Physiological trait compensations and the environment appeared to influence S_D_ and no QTLs were found. The confidence intervals surrounding QTLs were relatively large and fine mapping is needed to provide improved accuracy on physical locations, which would also provide clarity on the putative relationship between the identified QTLs and major loci.

## Figures and Tables

**Table 1:** Trait means for each parent (Tios and dic12b) and the tetraploid mapping population *(PS1)* individuals across the 2017 field trial and the 2019 pot trial. Generalised heritability (*H^2^*) is shown for each trait. Mean values in the table were calculated using raw data. Within each year, the asterisks by each trait name indicates significant differences at particular *p*-values between the mapping population parents (Tios and dic12b). The full test results are shown in Supplementary Tables S1 and S2.

2017 Field Trial					2019 Pot Trial				
Trait	Tios	dic12b	*PS1* ind	*H^2^*	Trait	Tios	dic12b	*PS1* ind	*H^2^*
A_L_ **	5.3	8.7	6.7	0.95	A_L_ **	5.6	7.5	5.9	0.90
F_T_ **	87.5	81.4	85.6	0.70	F_T_ **	89.8	77.5	83.8	0.77
FL_L_	24.1	25.6	24.0	-	FL_L_ **	18.1	25.8	20.4	0.64
FL_W_ **	1.9	1.4	1.6	0.36	FL_W_ *	1.6	1.5	1.5	0.59
S_D_ **	65.5	74.2	68.0	0.69	S_D_	72.4	67.2	75.9	0.36
T_D_ **	1.0	145.1	43.9	0.90	T_D_ **	0.3	121.9	26.6	0.72

A_L_ = awn length (cm); F_T_ = flowering time (days from sowing to anthesis); FL_L_ = flag leaf length (cm); FL_W_ = flag leaf width (cm); S_D_ = flag leaf stomatal density (mm^−2^); T_D_ = flag leaf trichome density (mm^−2^); *H^2^* = generalised heritability. - = *H^2^* was failed to be computed due to a negative variance term associated with genotype in the model. * = *p* < 0.05; ** = *p* < 0.01.

**Table 2:** Candidate quantitative trait loci (QTLs) identified using the tetraploid mapping population (*PS1*) for five traits, using phenotype data from two separate trials. Logarithm-of-odds (LOD) scores, percentage phenotypic variation explained by QTLs (% var) and additive effects were extracted from models fitted through the ‘fitqtl’ function in R/QTL. The interval shown for each QTL is the 1-LOD support interval expanded to the flanking markers. LOD thresholds are shown at the significance levels of 5% Alpha and 10% Alpha. These were determined through running 5000 permutations within the R/QTL ‘scanone’ function implemented through Haley–Knott regression [36].

QTL Name *	Chrom	cM	LOD	Interval (cM)	% var	Additive **	5% Alpha	10% Alpha
Q1_T_D__19^+^	1B	117.0	4.4	113.6–126.7	17.0	0.162^+^	4.34	4.01
Q2_FL_W__17	2B	148.0	5.0	142.7–156.2	15.8	−0.098	4.40	4.04
Q3_A_L__17	2B	183.9	5.0	69.0–193.5	12.3	0.860	4.31	3.98
Q4_A_L__19	4A	11.0	11.6	7.3–23.8	39.0	1.394	4.37	3.99
Q5_A_L__17	4A	15.1	12.2	9.3–28.4	35.1	1.539	4.31	3.98
Q6_FL_W__19	5B	206.7	4.5	201.2–215.4	17.4	0.090	4.31	3.98
Q7_FL_W__17	5B	226.5	6.1	222.5–229.7	19.7	0.045	4.40	4.04
Q8_FL_L__17^++^	6A	81.0	4.4	50.0–87.0	16.9	−1.75	4.34	3.98
Q9_F_T__17^++^	7B	13.0	7.1	4.0–23.0	25.7	−1.476	4.31	3.95
Q10_F_T__19	7B	20.0	4.7	4.0–27.3	18.2	−1.579	4.34	3.97

* Second and third elements of each QTL name signified the related trait and trial, respectively. ** Additive effect of 0.5 BB genotype (dic12b parent). ^+^ Data were expressed to the log10. ^++^ Residuals were used as trait data: the F_T_ 2017 data were corrected for germination heterogeneity and the FL_L_ 2017 data were corrected for F_T_.

**Table 3:** Genetic and physical positions of peak single nucleotide polymorphisms (SNPs) closest to quantitative trait loci (QTLs) identified using the tetraploid mapping population (*PS1*). Physical positions are also shown for the 1-LOD support interval markers. An NA indicated where an interval marker aligned to a different chromosome than the one mapped in the genetic map. Physical positions were taken from Basic Local Alignment Search Tool (BLAST+) results against RefSeq v1.0 wheat genome assembly [35].

QTL Name	Peak SNP	Chromosome	^G^ Position (cM)	^P^ Position (Mb)	Interval Start (Mb)	Interval Stop (Mb)
Q1_T_D__19	AX-94642880	1B	117.92	615.02	603.30	625.91
Q2_FL_W__17	AX-95247693	2B	147.31	558.04	539.81	576.09
Q3_A_L__17	AX-94664270	2B	183.91	680.41	58.85	711.72
Q4_A_L__19	AX-94933660	4A	10.51	47.14	40.44	102.62
Q5_A_L__17	AX-94941084	4A	15.13	79.05	47.11	109.84
Q6_FL_w__19	AX-94503623	5B	206.72	619.14	603.88	658.74
Q7_FL_w__17	AX-94402018	5B	226.49	671.30	662.65	NA
Q8_FL_L__17	AX-94416466	6A	82.53	518.79	74.50	535.89
Q9_F_T__17	AX-94622790	7B	17.05	13.83	6.15	23.44
Q10_F_T__19	AX-94622790	7B	17.05	13.83	6.15	29.31

## Supplementary Materials

The following are available online at https://www.mdpi.com/2223-7747/9/7/829/s1: Figure S1: Ear diversity within the *PS1* population and photos of the mapping parents, Figure S2: Frequency histograms of the six traits across both years, Table S1: Summary statistics and two sample t-test results using the mapping parent trait data, Table S2: Summary statistics and two-sample Wilcoxon test results using the mapping parent trait data. Table S3: The *PS1* genetic linkage map. Table S4: SNP marker data for the *PS1* population.
