# Supplementary material for: Identification of Quantitative Trait Loci Relating to Flowering Time, Flag Leaf and Awn Characteristics in a Novel Triticum dicoccum Mapping Population
Source: Plants (Basel). 2020 Jul 2;9(7):829. doi: 10.3390/plants9070829 (PMC7412379; doi:10.3390/plants9070829)
Supplement: Supplementary file 1 [file plants-09-00829-s001.zip › supplementary/Figure S1.pdf]

(a)

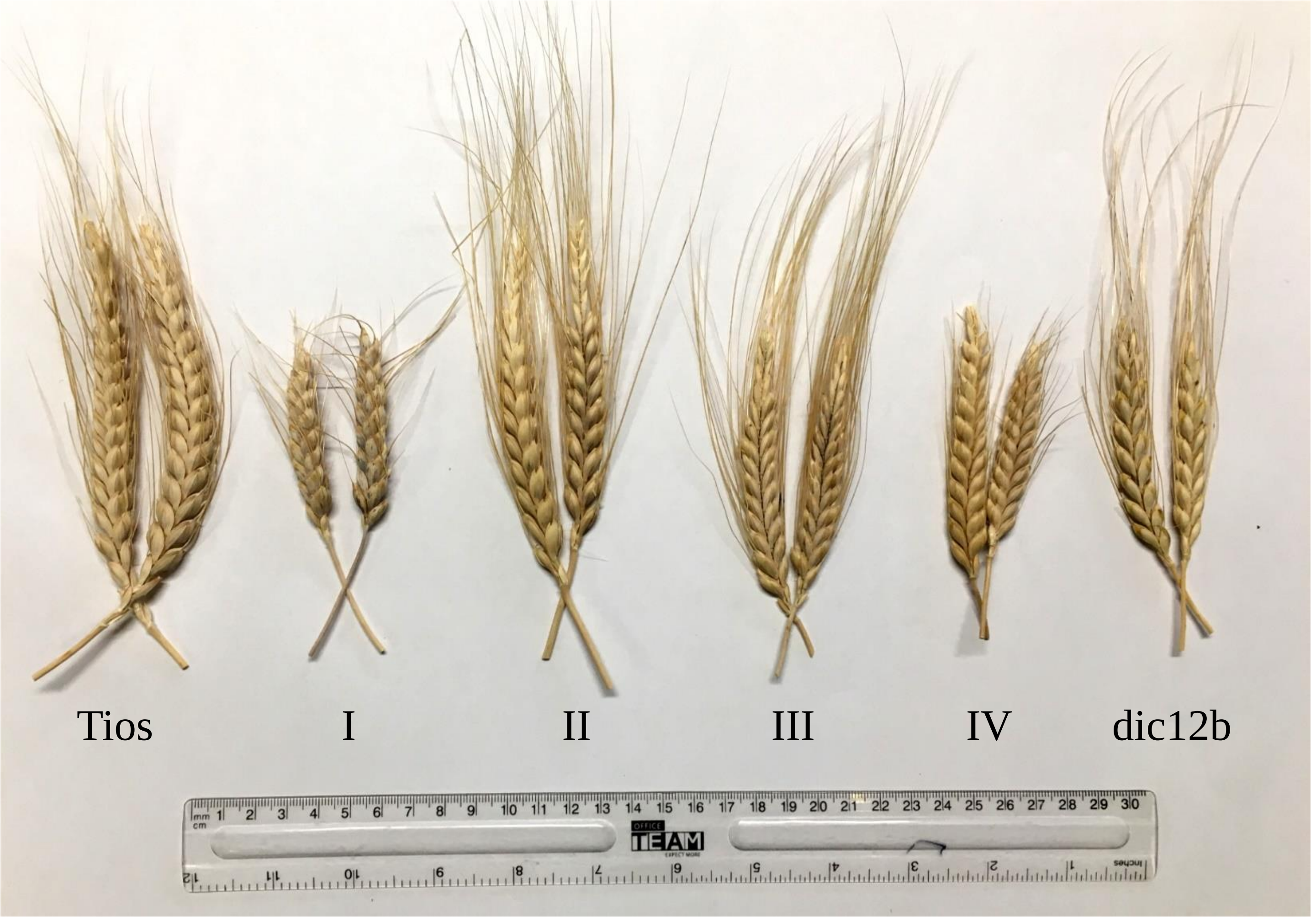

(b)

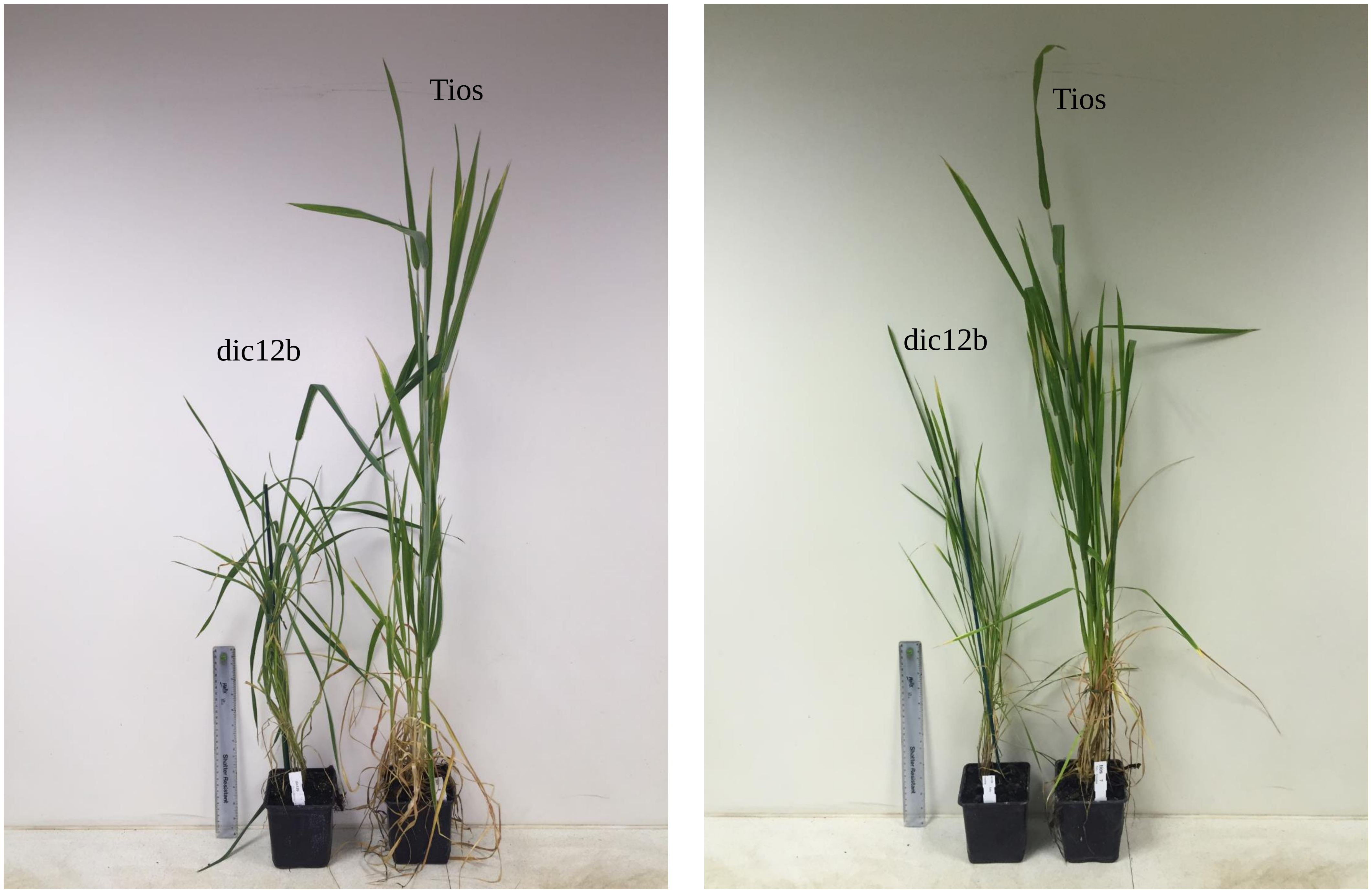

Supplementary Figure 1. (a) Ear diversity within the tetraploid mapping population (*PS1*); mature ears are shown from the mapping parents (Tios and dic12b) and four *PS1* individuals (labelled I - IV). ii). Two images showing dic12b and Tios plants during booting. A 30 cm ruler was included for scale.
