# Supplementary material for: Identification of Quantitative Trait Loci Relating to Flowering Time, Flag Leaf and Awn Characteristics in a Novel Triticum dicoccum Mapping Population
Source: Plants (Basel). 2020 Jul 2;9(7):829. doi: 10.3390/plants9070829 (PMC7412379; doi:10.3390/plants9070829)
Supplement: Supplementary file 1 [file plants-09-00829-s001.zip › supplementary/Figure S2.pdf]

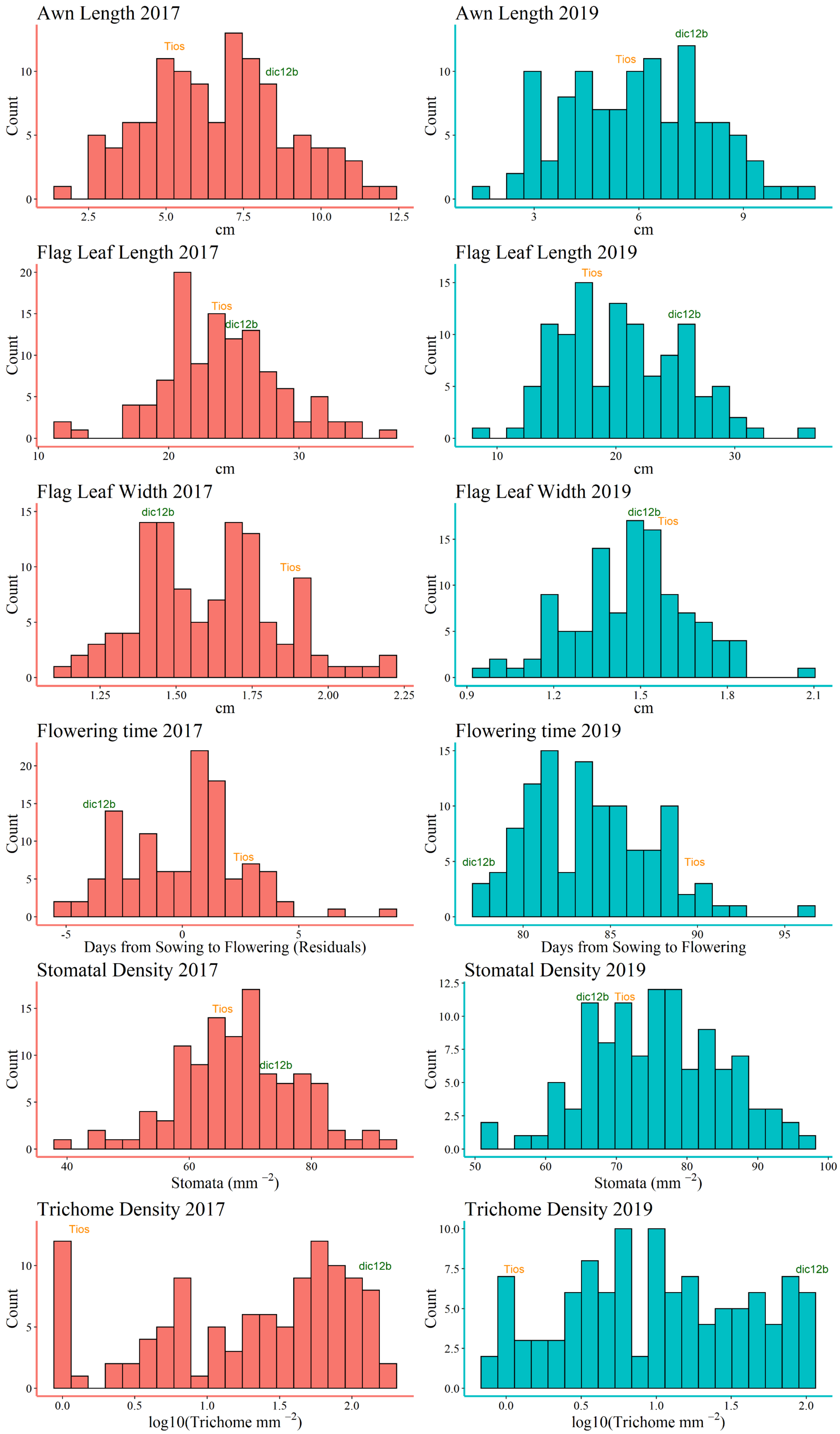

Figure S2. Frequency histograms of the six traits across both years. Mean parent values are shown for each trait with either a 'Tios' or 'dic12b' label. Plots were formed using the R package ggplot2 [61] and fonts were edited with the package extrafont [32].
