# Supplementary material for: Identification of Quantitative Trait Loci Relating to Flowering Time, Flag Leaf and Awn Characteristics in a Novel Triticum dicoccum Mapping Population
Source: Plants (Basel). 2020 Jul 2;9(7):829. doi: 10.3390/plants9070829 (PMC7412379; doi:10.3390/plants9070829)
Supplement: Supplementary file 1 [file plants-09-00829-s001.zip › supplementary/Table S1.pdf]

Table S1. Parent trait data from the 2017 field and 2019 pot trial, showing mean values, standard deviation and two sample t-test results for three traits. Results shown are from non-adjusted data, taken from both years.

	Tios		dic12b		Two Sample t-test		
Trait	*Mean*	*S.D*	*Mean*	*S.D*	*d.f*	*t*	*P*
$FL_{L\ 17}$	24.1	4.0	25.6	3.8	14	0.8	0.45
$FL_{L\ 19}$	18.1	3.2	25.8	4.7	38	6.0	<0.01
$A_{L\ 17}$	5.3	0.4	8.7	0.5	14	14.1	<0.01
$A_{L\ 19}$	5.6	0.5	7.5	0.7	38	10.0	<0.01
$S_{D\ 17}$	65.5	6.5	74.2	3.9	14	3.3	<0.01
$S_{D\ 19}$	72.4	7.8	67.2	10.9	38	-1.7	0.09

A_L = awn length (cm); FL_L = flag leaf length (cm); S_D = flag leaf stomatal density (mm^{-2}).
