# Supplementary material for: Identification of Quantitative Trait Loci Relating to Flowering Time, Flag Leaf and Awn Characteristics in a Novel Triticum dicoccum Mapping Population
Source: Plants (Basel). 2020 Jul 2;9(7):829. doi: 10.3390/plants9070829 (PMC7412379; doi:10.3390/plants9070829)
Supplement: Supplementary file 1 [file plants-09-00829-s001.zip › supplementary/Table S2.pdf]

Table S2. Parent trait data from the 2017 field and 2019 pot trial, where non-parametric tests were used to test for significant differences, showing mean and median values, standard deviation and two-sample Wilcoxon test results. Non-parametric tests were used when data for either parent were not normally distributed. Results shown are from non-adjusted data, taken from both years.

	Tios		dic12b		Tios	dic12b	Wilcoxon test	
Trait	*Mean*	*S.D*	*Mean*	*S.D*	*Median*	*Median*	*w*	*P*
$F_{T\,17}$	87.5	1.2	81.4	1.8	87	82	0	<0.01
$F_{T\,19}$	89.8	2.7	77.5	1.6	89	78	0	<0.01
$FL_{W\,17}$	1.9	0.2	1.4	0.2	1.9	1.4	3.5	<0.01
$FL_{W\,19}$	1.6	0.1	1.5	0.1	1.60	1.56	120.5	0.03
$T_{D\,17}^{*}$	0.1	0.3	2.2	0.1	0	2.2	64	<0.01
$T_{D\,19}^{*}$	0.04	0.2	2.1	0.2	0	2.1	400	<0.01

F_T = flowering time (days from sowing to anthesis); FL_W = flag leaf width (cm); T_D = flag leaf trichome density (mm^{-2}).

* = Data was expressed to the log10
